# High Fat-High Fructose Diet-Induced Changes in the Gut Microbiota Associated with Dyslipidemia in Syrian Hamsters

**DOI:** 10.3390/nu12113557

**Published:** 2020-11-20

**Authors:** Rachael G. Horne, Yijing Yu, Rianna Zhang, Nyan Abdalqadir, Laura Rossi, Michael Surette, Philip M. Sherman, Khosrow Adeli

**Affiliations:** 1 Cell Biology, Research Institute, Hospital for Sick Children, Toronto, ON M5G 0A4, Canada; rachael.horne@sickkids.ca (R.G.H.); philip.sherman@sickkids.ca (P.M.S.); 2 Molecular Medicine Programs, Research Institute, Hospital for Sick Children, Toronto, ON M5G 0A4, Canada; yijing.yu@mail.utoronto.ca (Y.Y.); rianna.zhang@sickkids.ca (R.Z.); nyan.abdalqadir@mail.utoronto.ca (N.A.); 3 Department of Laboratory Medicine and Pathobiology, University of Toronto, Toronto, ON M5S 1A8, Canada; 4 Department of Biochemistry and Biomedical Sciences, McMaster University Medical Centre, Hamilton, ON L8N 3Z5, Canada; rossil@mcmaster.ca (L.R.); surette@mcmaster.ca (M.S.); 5 Department of Paediatrics, University of Toronto, Toronto, ON M5S 1A8, Canada; 6 Department of Biochemistry, University of Toronto, Toronto, ON M5S 1A8, Canada; 7 Department of Physiology, University of Toronto, Toronto, ON M5S 1A8, Canada

**Keywords:** microbial metabolites, lipids, metabolism, metabolic dysfunction, 16S rRNA

## Abstract

**Aim:** The objective of this study was to characterize the early effects of high fructose diets (with and without high fat) on both the composition of the gut microbiota and lipid metabolism in Syrian hamsters, a reproducible preclinical model of diet-induced dyslipidemia. **Methods:** Eight-week-old male hamsters were fed diets consisting of high-fat/high-fructose, low-fat/high-fructose or a standard chow diet for 14 days. Stool was collected at baseline (day 0), day 7 and day 14. Fasting levels of plasma triglycerides and cholesterol were monitored on day 0, day 7 and day 14, and nonfasting levels were also assayed on day 15. Then, 16S rRNA sequencing of stool samples was used to determine gut microbial composition, and predictive metagenomics was performed to evaluate dietary-induced shifts in deduced microbial functions. **Results:** Both high-fructose diets resulted in divergent gut microbiota composition. A high-fat/high-fructose diet induced the largest shift in overall gut microbial composition, with dramatic shifts in the Firmicute/Bacteroidetes ratio, and changes in beta diversity after just seven days of dietary intervention. Significant associations between genus level taxa and dietary intervention were identified, including an association with *Ruminococceace NK4A214 group* in high-fat/high-fructose fed animals and an association with *Butryimonas* with the low-fat/high-fructose diet. High-fat/high-fructose feeding induced dyslipidemia with increases in plasma triglycerides and cholesterol, and hepatomegaly. Dietary-induced changes in several genus level taxa significantly correlated with lipid levels over the two-week period. Differences in microbial metabolic pathways between high-fat/high-fructose and low-fat/high-fructose diet fed hamsters were identified, and several of these pathways also correlated with lipid profiles in hamsters. **Conclusions:** The high-fat/high-fructose diet caused shifts in the host gut microbiota. These dietary-induced alterations in gut microbial composition were linked to changes in the production of secondary metabolites, which contributed to the development of metabolic syndrome in the host.

## 1. Introduction

Human gut microbiota, comprising of bacteria, viruses, phages, fungi and protists, play a profound role in mammalian health, contributing not only to host metabolism [1], but also to mucosal and systemic immune responses [2] and the metabolism of xenobiotics [3]. Numerous studies have found that the impact of the gut microbiota on host health relates to overall microbial composition and to bacterial diversity [4,5].

Over the last two decades, studies have focused on understanding the links between changes in the gut microbiota and the development of metabolic syndromes in humans, such as diabetes, obesity, and nonalcoholic fatty liver disease [1,6,7]. Both murine models and human studies reveal strong associations between alterations in the relative proportions of dominate gut phyla (that is, Bacteroidetes and Firmicutes) and obesity [8,9]. Changes in the gut microbiota are associated with host metabolic conditions, including changes in lipid profiles (such as low levels of high-density lipoprotein and increases in cholesterol and triglycerides) [10]. Recent work has determined one of the metabolic links between the gut microbiota and host cholesterol levels, with a microbial enzyme identified as a key factor in metabolizing dietary cholesterol, and subsequently limiting cholesterol absorption [11]. These findings indicate the critical role the gut microbiota play in maintaining host lipid homeostasis. Animal studies have found that varying levels of dietary fat and carbohydrates can have profound effects on the gut microbiota and host metabolism [12,13,14]. Specifically, a high-fat, high-sugar and low-fiber diet, commonly referred to as the “Western diet”, results in a shift in the gut microbiota composition, leading to increased lipopolysaccharide in circulation, causing pro-inflammatory responses that precede the development of insulin resistance and obesity [15]. The use of dietary interventions has also been explored as a means of correcting metabolic dysfunction in humans with decreased levels of triglycerides and low-density lipoproteins while improving microbial dysbiosis in nearly half of patients [16].

While previous studies reported links between diet manipulations, changes in the composition of gut microbiota, and subsequent metabolic disease, the underlying mechanisms are only now beginning to be delineated. Specifically, dietary manipulations have been found to impact levels of specific bacterial species within the gut microbiota, which are known to produce a range of bioactive metabolites, such as short-chain fatty acids, amino acids and vitamins [17].

Fructose, a common component of the western diet, has been shown to have profound effects on the liver, with high fructose diets shown to contribute to the development of nonalcoholic fatty liver disease [18]. High fructose diets have also been shown to cause shifts in the gut microbiota composition, reducing the abundance of beneficial *Bifidobacterium* and *Lactobacillus* [19]. In addition, high levels of fructose play a direct role in liver lipogenesis by increasing microbial short-chain fatty acid acetate [20], whereas diets high in fat reduce beneficial short-chain fatty acid butyrate [21]. In addition to the complex relationship between diet and microbial production of bioactive short-chain fatty acids, a bidirectional interaction between amino acids and gut microbiota has also been observed [22]. The gut microbiota aids in maintaining homeostasis of host amino acids by synthesizing several essential amino acids for the host, while microbial produced amino acids can also provide nutrients for the growth of amino acid-dependent commensal intestinal bacteria [23]. Diet composition influences microbial production of amino acids, with high-fat diets resulting in increases in microbial production of the branched-chain amino acids, i.e., isoleucine, valine and leucine [24], which are known to play an essential role in host lipid metabolism [25].

While much of the foundational work regarding how diet and the gut microbiota affect host metabolism has been performed in rodents, few studies have yet to be undertaken on Syrian hamsters. Importantly, Syrian hamsters have a reproducible response to dietary manipulation [26,27,28] and lipid metabolism that closely resembles that of humans [29]. Specifically, Syrian hamsters, like humans, but unlike mice and rats, have cholesterol ester transport protein activity [30], and respond to diets high in fat and cholesterol with the major plasma cholesterol being LDL-cholesterol (LDL-C) [26,27,28,31]. In addition to previous work on rodents, many previous studies have focused on the long-term effect of dietary manipulation, often evaluating the impact of months-long exposure to high fat and high carbohydrate diets [13,14,32,33]. In this study, we provided either a high-fat/high-fructose or low-fat/high-fructose diet to Syrian hamsters for a period of two weeks, in order to assess potential links between diet, gut microbiota, and host lipid metabolism in the early stages of metabolic derangement.

## 2. Materials and Methods

### 2.1. Animal Handling and Experimental Design

Syrian male hamsters with ~100 g body weight were purchased from Envigo (Mississauga, ON, Canada), acclimatized for one week after arrival and fed standard rodent chow (PicoLab rodent diet; catalog # 5058, St. Louis, MO, USA) prior to experimental manipulation. Hamsters were individually housed in bedded cages with access to environment enrichment objects. Cages were situated in a temperature-controlled room with a 12 h light-dark cycle. Animals were monitored closely for changes in body weight and baseline triglyceride levels prior to randomization. Animals were allowed free access to both food and water for the duration of the study protocol. The food from all diet groups was weighed every seven days to monitor consumption, and fresh chow was provided. Body weight was measured at baseline on day 0, and then again after 2 weeks of dietary intervention on experimental day 14. Food intake of individual hamsters was monitored weekly over the course of the experiment. This study was conducted in accordance with the Declaration of the Hospital for Sick Children, Toronto, ON, Canada, and the protocol approved by the Animal Care and Use Committee (project identification code #46136).

### 2.2. Experimental Diets

Hamsters were randomly divided into three groups (*n* = 9 per group) fed with varying diets for two weeks: (1) low-fat/high-fructose (HF) diet, (2) high-fat/high-fructose with 0.05% cholesterol (FFLC) diet, or (3) standard proprietary hamster diet (Chow) (PicoLab catolog #007689) (Table 1). Customized diets consisting of high sugar, high fat diet with 0.05% cholesterol (FFLC) (catalog # 180507) and 22% casein modified hamster diet with 60% fructose (HF) (catalog #161506) were purchased from Dyets Inc. (Bethlehem, PA, USA). A detailed comparison of experimental diet components can be found in Table 1 and Supplementary Table S1.

### 2.3. Fecal, Blood and Tissue Collection

On days 0, 7 and 14 after the start of dietary intervention, hamsters were fasted for 5 h before blood was drawn into lithium heparin-coated tubes via saphenous veins. Blood samples were centrifuged at 6000 rpm for 10 min at 4 °C, plasma was collected, and a mixture of protease inhibitors (SIGMAFAST™, Sigma-Aldrich, catalog # S8830-20TAB, St. Louis, MO, USA) added. All chemical assays were performed using plasma samples on the day of collection, with remaining plasma stored at −20 °C. Fecal samples were collected on concurrent days and stored at −80 °C. At day 15 post diet manipulation, hamsters were euthanized via exsanguination under isoflurane anesthetic by cardiac puncture. Liver and adipose tissues were then harvested, weighed and flash frozen.

### 2.4. Fasting Triglycerides and Cholesterol

Concentrations of plasma triglycerides (TG) and cholesterol (CH) were measured using commercial assay kits (ESBE-Randox, catalog # TR213, # CH200, respectively, Markham, ON, Canada). Briefly, 2 μl of each plasma sample plus 200 μl of reaction buffer were loaded in 96-well plates. After a 10 min incubation at room temperature, the colorimetric reactions were measured at a wavelength of 500 nm using a plate reader (Molecular Devices VersaMax 190, San Jose, CA, USA).

### 2.5. 16S rRNA Gene Sequencing

DNA was extracted from stool pellets, as previously described [34]. Sequences of the 16S rRNA gene variable 3–4 (V3–V4) regions were amplified using modifications previously described [34,35], and sequenced using the Illumina MiSeq platform. Primer and adaptor sequences were trimmed from the resulting sequences using Cutadapt [36]. DADA2 was used to filter and trim paired reads. Reads were discarded if the quality score was 2 or less, and if reads aligned to Phix human genome. DADA2 error correction was performed for each paired reads, de-noised reads merged, and any identified chimeric sequences then removed. Taxonomy was assigned to the resulting Amplicon Sequence Variants (ASVs) using RDP classifier trained with Silva v123 16S rRNA database [37]. Alpha and beta diversity analyses were performed on amplicon sequence variant (ASV) table using phyloseq and vegan packages in R v3.5.6. The predicted metagenomic function of the gut microbiota composition was performed using Phylogenetic Investigation of Communities by Reconstruction of Unobserved States (Picrust2) [38].

### 2.6. Statistical Analyses

Statistical analysis of 16S rRNA data was performed in R v3.5.6. Alpha diversity was assessed using linear regression, with diet and time as the fixed factors, animal ID as a random factor, and total reads per sample as an offset. Pairwise comparisons were assessed using the emmeans package in R, and multiple comparisons were corrected with Tukey’s post hoc adjustment. Negative binominal mixed model regression analysis was performed on the top 50 most abundant ASVs assigned to the genus level using NBZIMM r package (https://github.com/nyiuab/NBZIMM) [39]. Models were designed as follows: read counts of the selected ASV were used as the predicted variable, with dietary group and day used as the fixed factors, animal ID as a random factor, and the log of the total reads per sample used as an offset in the individual models. Models were evaluated using maximum likelihood testing and analysis of variance, significant interaction between dietary groups across time were evaluated by employing Tukey’s post hoc comparisons. Predicted microbial functional pathway abundance was normalized using total sum scaling, and the Kruskal Wallis test with False discovery rate (FDR) used for multiple comparison correction to assess changes in pathway abundance across days within treatment groups. Two-sided permutation Student’s t-test with FDR was used to assess differences in pathway abundance between groups at day 7 and day 14. Spearman correlation between microbial abundance and metabolic biomarkers was performed using a center log ratio transformed ASV table filtered to retain the top 25 ASVs with genus level taxonomy, and then corrected for multiple comparisons using FDR. The same approach was used for correlation of predicted pathway abundance; however, analysis was restricted to the top 200 abundant pathways. All other statistical analyses were performed using GraphPad prism software (GraphPad, San Diego, CA, USA), using one-way and two-way ANOVA where appropriate with Tukey’s post hoc testing. Data normality was assessed by Shapiro-Wilk test and results are presented as means, ±SEM.

## 3. Results

### 3.1. High-Fructose Diets Do Not Have a Significant Impact on the Weight Gain of Hamsters

**Figure 1:**
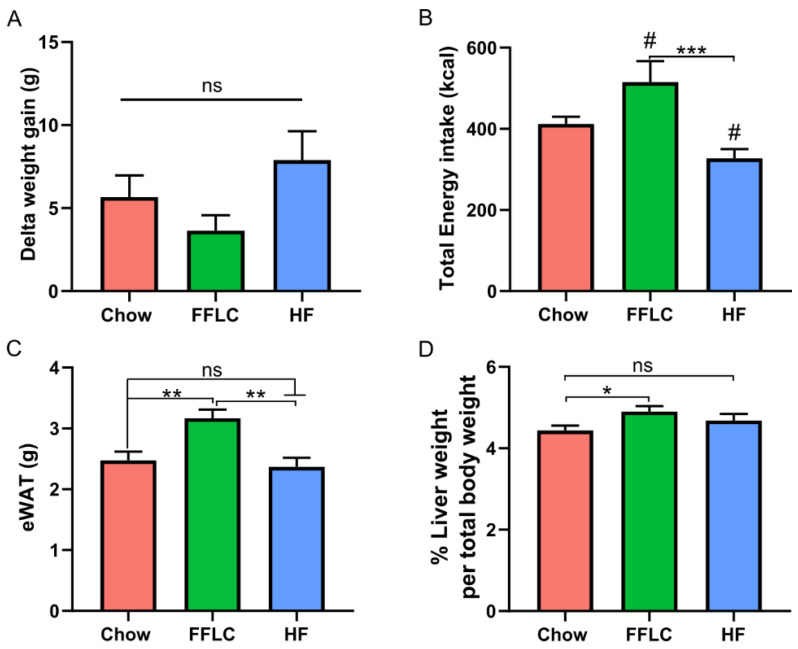
High-fat/high-fructose diet results in increased adiposity and hepatomegaly without weight gain after 14 days. (**A**) Body weight gain across 2 weeks. (**B**) Total gross energy intake over the two-week study period (kcal); # denotes no significant difference when compared to control chow. (**C**) Epididymal white adipose tissue (eWAT) weight. (**D**) Percent liver weight per total body weight measured at study day 14. Values are expressed as means, ±SEM. Significance denoted as * *p* < 0.05, ** *p* < 0.01, *** *p* < 0.001 vs control chow using one-way ANOVA, with Tukey’s multiple comparison test. FFLC denotes high-fat/high-fructose diet, HF indicates low-fat/high-fructose diet intervention, ns indicated no significance.

Hamsters were fed with either low-fat/high-fructose, high-fat/high-fructose, or control diet (chow) for two weeks, and the body weight of each hamster was measured before and after the two-week dietary intervention. No significant difference in body weight was found between animals assigned to the three dietary treatment groups on day 0 (data not shown). After 14 days of dietary intervention, no significant difference in body weight gain was observed among the three dietary groups (Figure 1A). In addition, we evaluated total gross energy intake over the two-week experimental period and found no significant difference in energy intake between experiment diets when compared to control diet; however, animals fed with high-fat/high-fructose diet exhibited an increase in energy intake compared to low-fat/high-fructose fed hamsters (Figure 1B).

### 3.2. High-Fat/High-Fructose Diet Causes Hepatomegaly and Increased Adipose Tissue

To determine the effects of dietary fat and fructose on the accumulation of body fat and the development of hepatomegaly, we measured epididymal white adipose tissue weight (eWAT) and found that hamsters fed a high-fat/high-fructose diet developed significantly higher amounts of eWAT compared to animals fed the control diet (*p* < 0.05), whereas the low-fat/high-fructose diet feeding group did not affect eWAT (Figure 1C). The high-fat/high-fructose diet also significantly increased the ratio of liver weight to total body weight (Figure 1D). By contrast, there was no significant alteration in liver weight in the low-fat/high-fructose diet fed animals compared to those on the chow control diet (*p* > 0.05).

### 3.3. High-Fat/High-Fructose Diet Induces Dyslipidemia

**Figure 2:**
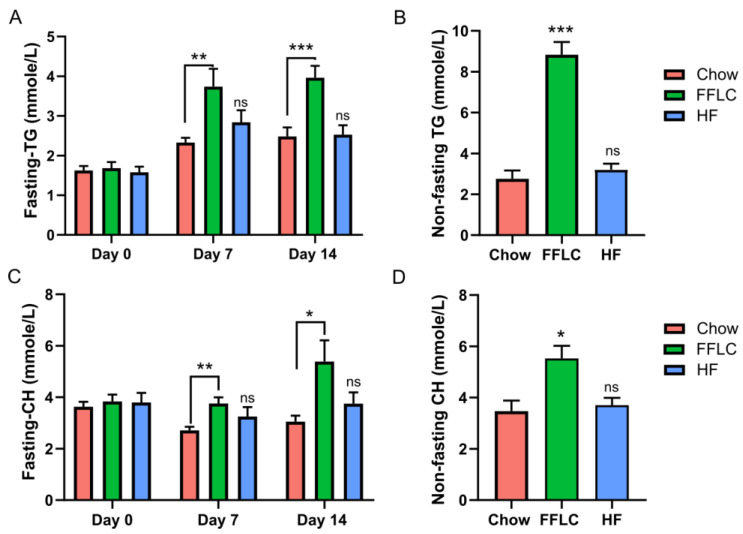
High-fat/high-fructose diet induces dyslipidemia. (**A**) 5 h fasting plasma TG on day 0 (*n* = 6), day 7 (*n* = 6), and day 14 (*n* = 9); (**B**) nonfasting plasma TG; (**C**) Fasting plasma total CH on study day on day 0 (*n* = 6), day 7 (*n* = 6), and 14 (*n* = 9); (**D**) Nonfasting plasma total CH on day 15 (*n* = 9). Values are expressed as means, ±SEM. Significance denoted as * *p* < 0.05, ** *p* < 0.01, *** *p* < 0.001 vs Chow, using Dunnett’s multiple comparison test. TG denotes triglycerides, CH cholesterol, FFLC indicates a high-fat/high-fructose diet, and HF a, low-fat/high-fructose diet, ns denotes no significant difference.

To evaluate the impact of diet on lipid metabolism, we measured fasting levels of triglycerides and cholesterol in plasma collected at baseline (day 0), day 7, and day 14 post-dietary treatments. The high-fat/high-fructose diet resulted in a significant increase of fasting triglycerides by day 7 (*p* < 0.05), which remained elevated at day 14 (*p* < 0.001) (Figure 2A). We also measured the nonfasting level of triglycerides on day 15; elevated triglycerides were found only in the high-fat/high-fructose group (Figure 2B). Similar effects were observed for fasting levels of cholesterol: hamsters fed with a high-fat/high-fructose diet exhibited increased levels of cholesterol at day 7 and 14, compared to the control diet fed group (*p* = 0.01 and *p* = 0.04, respectively) (Figure 2C). By contrast, there were no significant differences noted in the low-fat/high-fructose fed animals at either time point (*p* > 0.05), when compared to control chow fed study group. Additionally, the level of nonfasting cholesterol measured on day 15 post diet manipulation was elevated only in high-fat/high-fructose fed group of hamsters (*p* < 0.01), compared to control chow fed group (Figure 2D). Thus, we found that a high-fat/high-fructose diet induces dyslipidemia in hamsters, whereas a high-fructose/low-fat diet does not.

### 3.4. High-Fat/High-Fructose and Low-Fat/High-Fructose Diets Alter the Intestinal Microbiota

**Figure 3:**
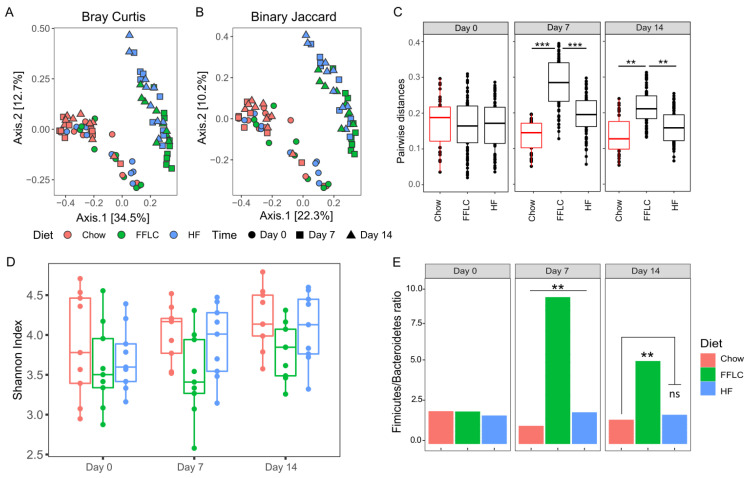
Feeding of either a high-fat/high-fructose or low-fat/high-fructose diet for 14 days leads to shifts in intestinal microbial composition and changes in dominant bacterial Phyla. (**A**) Principle coordinate analysis by Bray-Curtis similarities distances; (**B**) Principle coordinate analysis by Binary Jaccard index, significance assessed using repeated measure (PERMANOVA); (**C**) Pairwise dissimilarity distance comparison between dietary groups on day 0, day 7, and day 14 using Unweighted Unifrac distances, one-way ANOVA followed by Tukey’s multiple comparison testing; (**D**) Alpha diversity measured by Shannon index, two-way ANOVA, with total read depth used as a model offset, Tukey’s multiple comparison testing; and (**E**) Firmicutes/ Bacteroidetes ratio, two-way ANOVA, Tukey’s multiple comparison testing; ** *p* < 0.01, *** *p* < 0.001, ns denotes no significance, FFLC denotes a high-fat/high-fructose diet, HF a low-fat/high-fructose diet and ASV denotes amplicon sequence variant.

To understand the underlying mechanisms by which the diets contribute to the regulation of lipid metabolism, we investigated the effects of dietary manipulations on the composition of the gut microbiota. As measured by beta diversity, there was a significant difference in overall microbial composition between dietary groups. This divergent microbial composition was highly variable at each time point, as there was a significant interaction between dietary group and treatment day when using two distinct repeated measure tools for the analysis of beta diversity, Bray Curtis (*p* < 0.05; Figure 3A) and Binary Jaccard (*p* < 0.05; Figure 3B). Pairwise comparisons of dietary treatment groups with control chow diet revealed that after seven days of dietary intervention, hamsters fed the high-fat/high-fructose diet exhibited the greatest shift in the composition of gut microbiota (*p* < 0.0001). On day 14 post dietary manipulation, pairwise comparisons indicated that the high-fat/high-fructose diet group maintained the microbiota compositional change, whereas the difference in dissimilarity distance between low-fat/high-fructose and chow fed animals was reduced (Figure 3C). No significant differences were found within the control group across the three time points using both Binary Jaccard and Unweighted Unifrac metrics (*p* > 0.05).

No significant differences in alpha diversity between the dietary groups were found, as measured by Shannon index between day 0 to day 14 (*p* > 0.05) (Figure 3D). A trend towards decreased alpha diversity was observed on day 7 for high-fat/high-fructose diet fed hamsters, compared to control diet (*p* = 0.06). However, there were no significant differences when comparing within dietary treatment groups across sampling days (*p* > 0.05).

In addition to overall microbial composition, gut microbiota was evaluated at the phylum level. We observed that the Firmicutes/Bacteroidetes ratio differed significantly between dietary groups and across days of intervention: high-fat/high-fructose fed animals exhibited a significant increase in Firmicutes between day 0 and day 7 (58% versus 87% respectively, *p* = 0.0042; Figure 3E). Between day 7 and day 14, the Firmicutes/Bacteroidetes ratio was decreased in high-fat/high-fructose diet fed hamsters, although the ratio remained elevated compared to standard chow and low-fat/high-fructose diet fed groups (Figure 3E). By contrast, there was no significant change in the Firmicutes/Bacteroidetes ratio for either the control diet or low-fat/high fructose diet groups from day 7 to day 14 (*p* > 0.05). Evaluating clinically relevant phyla Proteobacteria, we found no significant changes in any of the dietary treatment groups (*p* > 0.05).

### 3.5. High-Fat/High-Fructose and Low-Fat/High-Fructose Diets Induce Changes in Bacterial Taxa of Gut Microbiota

**Figure 4:**
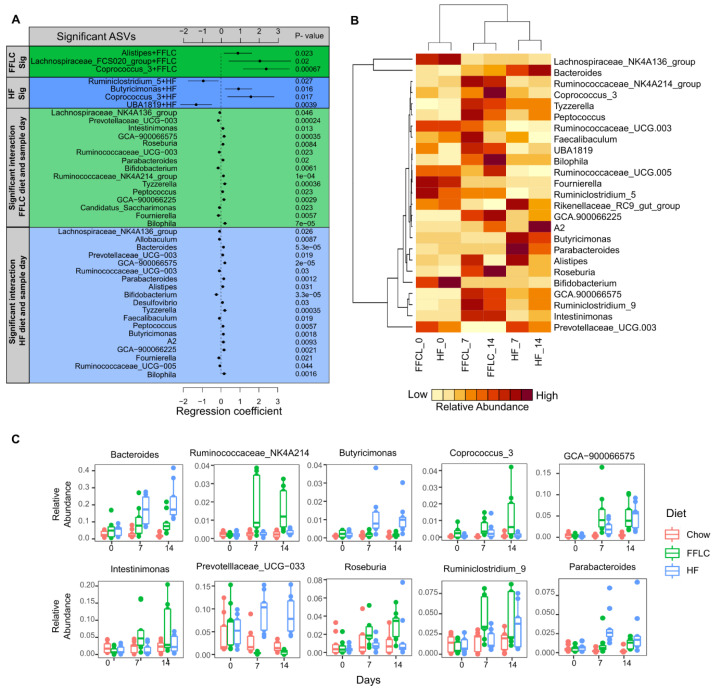
Dietary interventions over 14 days result in differential association of bacterial taxa abundance at the genus level. (**A**) Negative binomial regression coefficients for significant genus level ASVs associated with dietary interventions and changes across time. Dots represent coefficient and bars represent standard error of the estimate; (**B**) Heat-map of the relative abundance of bacterial taxa with significant interactions between dietary group and time; (**C**) Relative abundance of bacterial taxa with divergent responses to either study diet across two weeks of intervention. High-fat/high-fructose diet is indicated by FFLC, HF denotes low-fat/high-fructose (HF) diet intervention and ASV denotes amplicon sequence variant.

Negative binomial regression modeling identified amplicon sequence variants (ASVs) at the genus level associated with each individual dietary group and the temporal relationship across the two-week intervention period. As shown in Figure 4A, three ASVs were positively associated with the high-fat/high-fructose diet: *Alistipes, Lachnospiraspeae FCS020,* and *Coprococcus*. Low-fat/high fructose fed hamsters exhibited a positive association with *Coprococcus*, a unique positive association with *Butyricimonas*, and a negative association with *Ruminoclostridum 5* and *UBA 1819*.

A total of 25 taxa were identified with a significant interaction between dietary group assignment and time measured in days (Figure 4B). Of these 25 taxa, ten were differentially affected between high-fat/high-fructose and low-fat/high-fructose diet fed hamsters (Figure 4C). Notably, a significant increase in *Ruminococceace NK4A214 group, Intestinimonas, Roseburia* and *Ruminiclostridum 9* was observed in high-fat/high-fructose diet fed hamsters, whereas low-fat/high-fructose diet fed animals exhibited increases in *Bacteroides, Butyricimonas, Parabacteroides* and *Prevotella*. Interestingly, *Prevotella* responded divergently in the two dietary groups: decreasing in the high-fat/high-fructose diet fed animals while increasing in those receiving the low-fat/high-fructose diet.

### 3.6. Dietary-Induced Shifts in Gut Microbiota Composition Correlate with Triglyceride and Cholesterol Levels

**Figure 5:**
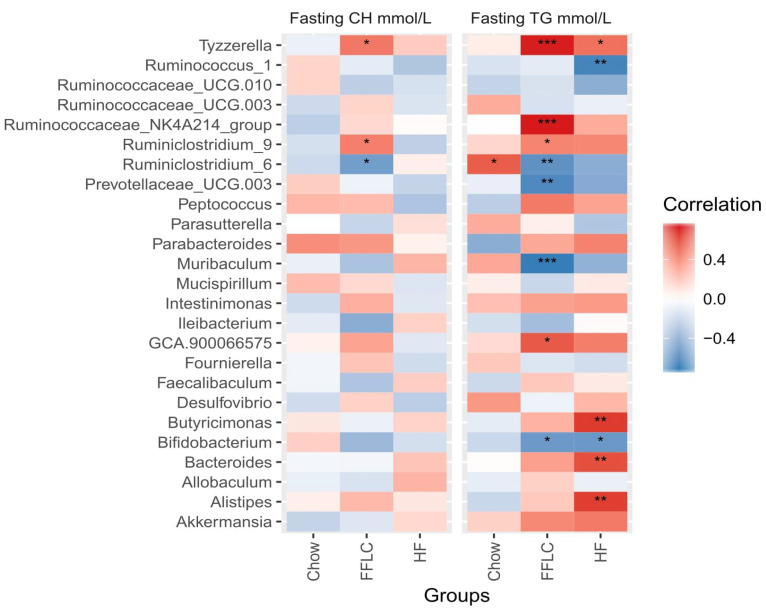
Correlation heatmap representing Spearman correlation coefficients between changes in center log ratio normalized abundance of bacterial taxa (at the genus level) and the lipid profile biomarkers fasting CH and fasting TG within dietary treatment groups. Significance denoted as * padj < 0.05, ** padj < 0.01, *** padj < 0.001. CH denotes cholesterol, TG denotes triglyceride, and FFLC denotes a high-fat/high-fructose diet, and HF a low-fat/high-fructose diet.

We observed that high-fat/high-fructose diet induced both dyslipidemia and alterations of gut microbiota in hamsters, and further analyzed whether there was a correlation between these two parameters. As shown in Figure 5, three ASVs that significantly correlated with fasting cholesterol levels were unique to the high-fat/high-fructose diet fed hamsters: *Ruminiclostridium 9* (*ρ* = 0.45) *Tyzzerella* (*ρ* = 0.48) and *Ruminiclostridium 6* (*ρ* = −0.53). No significant correlations between genus level taxa and fasting levels of cholesterol were found in the low-fat/high-fructose diet fed group of animals.

When analyzing fasting levels of triglyceride in plasma, we observed that the high-fat/high-fructose diet fed study group had a total of eight significant correlations, with four positive correlations, i.e., *Tyzzerella* (*ρ* = 0.73)*, Ruminococceace NK4A214 group* (*ρ* = 0.73)*,* GCA.900066575 (*ρ* = 0.58) and *Ruminiclostridium 9* (*ρ* = 0.44), and four negative correlations, i.e., *Muribaculum* (*ρ* = −0.71), *Prevotellaceae_UCG.003* (*ρ* = −0.64), *Bifidobacterium* (*ρ* = −0.55) and *Ruminiclostridium 6 (ρ* = *−0.59).* Paradoxically, within the low-fat/high-fructose group, opposite correlations were observed in the *Ruminiclostridium* genus: *Ruminiclostridium 6* exhibited a negative association with plasma levels of cholesterol and triglycerides, whereas *Ruminiclostridium 9* presented a positive correlation with these two lipid metabolism markers.

Two taxa were identified as having similar correlations with triglycerides in both dietary intervention groups, with hamsters fed a low-fat/high-fructose diet exhibiting almost identical correlation with *Bifidobactrium* (*ρ* = −0.56) and *Tyzzerella (ρ* = 0.51) compared to the high-fat/high-fructose diet fed group. These findings indicate that the alterations in these taxa may relate to triglyceride levels regardless of specific dietary manipulations.

The low-fat/high-fructose diet group exhibited four unique correlations with fasting levels of triglyceride, including three positive correlations: *Butyrimonas* (*ρ* = 0.67), *Bacteroides* (*ρ* = 0.61), *Alistipes* (*ρ* = 0.65), and one negative correlation with *Ruminococcus 1* (*ρ* = −0.67).

### 3.7. Dietary Intervention Impacts Functional Changes in the Gut Microbiota

**Figure 6:**
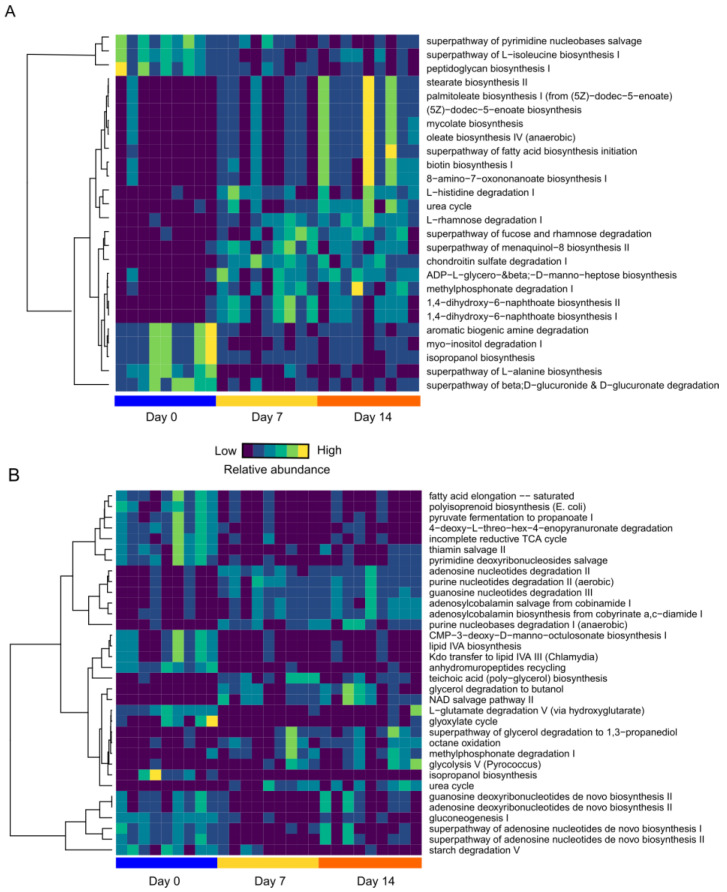
Predicted metagenomic changes within dietary intervention groups across sampling days. (**A**) Metagenomic pathways significantly changed across treatment days for the low-fat/high-fructose fed group of hamsters; and (**B**) Metagenomic pathways significantly changes across treatment days in the high-fat/high-fructose fed group of animals. Kruskal Wallis test, with multiple comparison corrections with FDR. Cytosine monophosphate, CMP; Tricarboxylic acid cycle, TCA; nicotinamide adenine dinucleotide, NAD; Adenosine diphosphate, ADP.

In addition to the microbial composition, an investigation was conducted to determine whether low-fat/high-fructose or high-fat/high-fructose diets had any impact on the function of gut microbiota. In the low-fat/high-fructose diet fed hamsters, 26 pathways were notably changed, which included increases in fucose and rhamnose degradation, and biosynthesis of fatty acids (e.g., oleate and mycolate) (Figure 6A). Interestingly, a shift toward increased energy production was observed, with increases in the electron transport chain including 1,4-dihydroxy-6-napthoate biosynthesis, and menaquinol-8 biosynthesis II. An increase in the biosynthesis of the important cofactor biotin was also observed in low-fat/high-fructose fed animals. Previous studies have reported that biotin affects lipid and carbohydrate metabolism [40] and reduces both hyperglycemia and hypertriglyceridemia [41].

In the high-fat/high-fructose diet fed group, a total of 34 pathways were significantly altered from day 0 to day 14 (Figure 6B). Notably, there was a shift towards catabolism of nucleotides, as evidenced by a reduction in nucleotide biosynthesis accompanied by an increase in nicotinamide adenine dinucleotide (NAD), thiamine and vitamin B12 salvage pathways. Fatty acid elongation, lipid biosynthesis, and gluconeogenesis were also decreased. Both high-fructose diets resulted in significant increases in urea cycle pathways from day 0 to day 14, likely resulting from an increase in available energy capacity.

The comparison of gut microbiota metabolic pathways affected by the low-fat and high fat with high-fructose diets on day 7 and day 14 revealed an increase in amino acid biosynthesis pathways, including lysine, arginine, glutamate, glutamine and the branched-chain amino acid isoleucine in the microbiome of high-fat/high-fructose diet fed animals. This predictive metagenomic analysis also suggested that the low-fat/high-fructose diet group had an increase in fatty acid elongation and gluconeogenesis, accompanied by an increase in vitamin B cofactor biosynthesis, including tetrahydrofolate and thiamine (Supplemental Figure S1).

### 3.8. High-Fat/High-Fructose Diet Leads to Functional Changes in the Gut Microbiome which Contribute to Host Dyslipidemia

For hamsters fed a high-fat/high-fructose diet, a total of 22 pathways significantly correlated with metabolic markers: 19 with fasting levels of triglycerides (15 negatively correlated and four positively correlated) and three with fasting levels of cholesterol (Table 2 and Table 3). Interestingly, all four pathways that positively correlated with fasting levels of triglyceride in hamsters receiving a high-fat diet were related to nucleotide degradation. Notably, a negative correlation was observed between fasting levels of triglycerides and pyruvate fermentation for propionate in hamsters on a high-fat/high-fructose diet. Propionate has previously been shown to regulate triglyceride levels through expression of the peroxisome proliferator-activated α receptor [42]. Moreover, there was a significant negative correlation between aspartate and asparagine biosynthesis pathways and levels of both triglycerides and cholesterol in hamsters fed a high-fat/high-fructose diet.

## 4. Discussion

This study uncovers the early impacts of dietary fat and carbohydrates on the gut microbiota and metabolic profiles of golden Syrian hamsters. Feeding either high-fat/high-fructose or low-fat/high-fructose diets resulted in shifts in overall composition of the gut microbiota, with high-fat/high-fructose diet resulting in more profound changes in both the gut microbiota and lipid metabolic profiles compared to a low-fat/high-fructose diet. A few previous studies have also employed this model to determine how diet can impact the composition of gut microbiota [43,44,45,46,47]. However, most of these reports assessed the addition of a single dietary component over a longer dietary intervention time to determine the resulting effects on host lipid metabolism [43,44,45,46,47], thereby overlooking earlier metabolic changes.

The high-fat/high-fructose diet, which more closely resembles the so-called “Western” diet [48], elevated plasma levels of triglycerides and cholesterol within just seven days. By contrast, a low-fat/high-fructose diet did not result in a significant increase in host circulating lipid levels. Previous work has described the lipogenic nature of fructose [49,50], and the synergistic effects of fructose and high fat [14,51]. In the current study, we found that low-fat/high-fructose diet did not induce dyslipidemia after two weeks, which is consistent with studies by Lozano et al, reporting that the addition of fructose to a normal diet did not produce any metabolic changes in rats, whereas the combination of high-fructose with high-fat resulted in metabolic changes greater than those observed with a high-fat diet alone [14]. Notably, we did not see any significant difference in gained body weight after the two-week intervention between the three diets. The lack of weight gain observed in both fructose diets, also observed in several other studies [19,52,53], may be a result of the thermogenic effect of fructose, previously observed in both humans [54,55] and rodents [56]. We also observed significant increases in adiposity and liver weight only in high-fat/high-fructose fed hamsters. These findings are in contrast with our previous work demonstrating an impact of fructose feedings on host lipid profiles [50,57]. However, previous studies did not evaluate the role of the gut microbiota in contributing to the host response to dietary interventions.

In this study, we found that the combined high-fat and high-fructose diet resulted in a dramatic shift in the composition of gut microbiota. Specifically, seven days of dietary intervention resulted in a significant shift in microbial composition, which was still evident at day 14, albeit to a lesser degree. This may suggest a transient change in microbiota was induced by high-fat/high- fructose diet and that the microbiota profile was under recovery after 14-days of feeding. Such a finding has been observed in previous reports with various diets and oral antibiotics [58,59,60]. Interestingly, there were no marked changes in overall diversity of commensal bacterial species, indicating that changes in the composition of the gut microbiota resulted primarily from shifts in the abundance of bacterial species that were previously presented at lower levels. The Firmicute/Bacteroidetes ratio, a metric commonly associated with both obesity and metabolic dysfunction [61], was found to be altered only in the high-fat/high-fructose diet. We also observed unique changes at the genus level that were significantly associated with dietary interventions. For instance, high-fat/high-fructose diet increased the abundance of *Ruminococceace NK4A214 group*, *Intestinimonas*, *Roseburia* and *Ruminiclostridium 9*, each of which has previously been linked to obesity [62,63,64]. In addition, we found an increase in the abundance of *Parabacteroides,* a genus with anti-inflammatory properties [65], in the low-fat/high-fructose diet fed group. Recent work notes that *Parabacteroides distasonis* alleviates obesity in mice fed with a high-fat diet via regulating the succinate and secondary bile acid metabolism [66]. The increased abundance of *Parabacteroides* observed only in the low-fat/high-fructose diet group of hamsters may explain the lack of demonstrable host lipid responses to dietary intervention.

Previous studies using germ-free mice showed that host lipid profiles can be transferred via conventionalization of the intestinal microflora [67]. The phenomenon also has been observed in humans following fecal microbial transplantation [68]. We observed a comparable relationship by evaluating correlations between changes in microbial abundance at the genus level and fluctuations in host lipid profiles. Several notable correlations were identified, including a positive correlation between plasma levels of triglycerides and cholesterol with *Tyzzerella*, a Gram-negative member of the Lachnospireacae family, in the gut. Recent work indicates that the abundance of *Tyzerella* is a risk factor related to the development of cardiovascular diseases [69]. Correlations were also found in the present study between *Ruminiclostridium* and host lipid levels, which is of interest given previous work linking *Ruminiclostridium* with obesity [64] and gestational diabetes [70]. Notably, *Ruminiclostridium 6* was previously found to have a strong positive correlation with ghrelin levels, indicating a potential role in mediating the hunger response and resulting changes in energy intake [71].

Correlations between microbial abundance and metabolic markers reveal a relationship between gut microbial taxa and host metabolism. However, this approach alone provides little information regarding underlying mechanisms. By evaluating functional shifts related to changes in the gut microbiota between various dietary interventions, we uncovered additional details about potential mechanistic contributions to host dyslipidemia via the production of secondary metabolites, although validation studies employing shotgun metagenomic sequencing and metabolomics are warranted.

In the current study, the metabolic pathway of biotin biosynthesis was increased across treatment days in hamsters fed a high-fructose/low-fat diet. A previous study demonstrated that the treatment of biotin in mice protects against metabolic syndrome induced by a diet high in fructose [72]. The increase in biotin by gut microbes following the high-fructose feedings observed here may serve as a compensatory response, accounting for the lack of increase in plasma lipids in hamsters fed with a low-fat/high-fructose diet. Notably, we observed an increase in urea cycle for both high-fructose/high-fat and high-fructose/low-fat diets. Previous findings have shown an effect of fructose feeding on liver uric acid production [73], which is known to contribute to hepatic injury and systemic inflammation [74]. Our findings indicate that the gut microbiome may also be a contributing factor for increased circulating levels of uric acid following fructose feedings.

Low-fat/high-fructose diet fed hamsters also exhibited an increase in levels of the butyrate-producing bacterium, *Butryimonas* [75]. Butyrate levels are linked to improved metabolite outcomes both in humans and in animal studies, by acting as a regulator of adipocytes [76,77]. Butyrate supplementation has also been found to improve the dyslipidemia caused by a high-fat diet in mice [78]. While not measured directly in the current study, an increase in levels of butyric acid in the low-fat/high-fructose diet fed group is one potential mechanism that could account for the lack of host dyslipidemia, as well as the lack of increase in epididymal adipose tissue observed herein.

By contrast, high-fat/high-fructose fed hamsters exhibited a decrease in fermentation pathways that control production of the short-chain fatty acid, propionate. In humans, propionic acid stimulates glucagon-like peptide-1 and pancreatic polypeptide [79], and long-term delivery of propionate into the colon reduces weight gain and intra-abdominal fat accretion in overweight adults [79]. Changes in this pathway in high-fat/high-fructose diet fed hamsters correlated with fasting levels of triglycerides. This relationship is likely a reflection of the effects of propionate on host lipid metabolism. Propionate is known to directly affect lipid metabolism by decreasing the enzymatic activities of 3-hydroxy-methlyglutaryl-CoA synthase and reductase [80], critical enzymes in cholesterol synthesis [81] and ketogenesis [82]. Additionally, propionate regulates triglyceride production through increasing expression of PPAR-α receptor, which subsequently leads to an increase in β-oxidation and a reduction in triglycerides [42]. Other short-chain fatty acids also affect PPAR-γ receptor expression [83] and β-oxidation in colonocytes. This relationship between microbial production of short-chain fatty acids and host energy metabolism is critical in maintaining a healthy homeostatic relationship between the host and constituents of the gut microbiota [84].

In addition to changes in the biosynthesis of short-chain fatty acids, there were negative correlations between amino acid (aspartate and asparagine) biosynthesis and fasting metabolic parameters in the high-fat/high-fructose diet group. Previous studies in humans indicate that plasma levels of asparagine are associated with a lower risk of type-2 diabetes [85]. Asparagine levels are inversely correlated with levels of plasma lipids. Moreover, feeding of exogenous L-aspartate has been shown to limit the progression of fatty liver in cholesterol-fed rabbits [86]. The high-fat/high-fructose diet also induced a significant increase in production of branched-chain amino acid isoleucine, which is of potential importance since high levels of circulating isoleucine are associated with inactivity [87], type 2 diabetes [88], and metabolic syndrome [89].

Taken together, these findings suggest that a two-week period of dietary intervention with a high-fat/high-fructose diet not only induces changes in gut microbial composition, but also affects microbial metabolic functions, which have the potential to produce metabolites contributing to host dyslipidemia and development of the metabolic syndrome. An improved understanding of the complex relationships between diet, the gut microbiota, and host metabolism provides clinicians and researchers with the knowledge needed to improve outcomes in humans affected by metabolic diseases. Herein, we identify several key taxa and microbial metabolic pathways that are influenced by fructose and fat dietary supplementation. To further evaluate the specific relationship between dietary fats and fructose, future studies should evaluate the impact of high fat, low fructose diets using the same animal model. Here, we employed 16S rRNA sequencing and predictive metagenomics; while these methods yield robust preliminary findings, further validation is required using shotgun metagenomic and metabolomic evaluations.

## Figures and Tables

**Table 1:** Dietary protein, fat, carbohydrate, and total energy of each diet employed in the current study.

	kcal/kg			g/kg		
	Chow	FFLC	HF	Chow	FFLC	HF
**Protein**	872	613	796	218	171	222
**Fat**	819	2700	540	91	300	60
**Carbohydrate**	2072	1572	2280	518	414	600
**Gross Energy**	4600	4971	3655			

**Table 2:** Significant correlations between bacterial metabolic pathway abundance and fasting triglycerides.

Predicted Metabolic Pathways	ρ	Diet	AdjP
Superpathway of l-alanine biosynthesis	−0.59	FFLC	0.011
l-histidine degradation I	−0.59	FFLC	0.012
Anhydromuropeptides recycling	−0.56	FFLC	0.019
Superpathway of l-aspartate and l-asparagine biosynthesis	−0.54	FFLC	0.024
Pyruvate fermentation to propanoate I	−0.54	FFLC	0.025
UDP N-acetylmuramoyl pentapeptide biosynthesis II lysine containing	−0.52	FFLC	0.032
Peptidoglycan biosynthesis III mycobacteria	−0.52	FFLC	0.034
Peptidoglycan biosynthesis I meso diaminopimelate containing	−0.51	FFLC	0.037
CMP 3 deoxy d-manno-octulosonate biosynthesis I	−0.50	FFLC	0.042
Superpathway of geranyl-geranyl diphosphate biosynthesis II via MEP.	−0.50	FFLC	0.044
Starch degradation V	−0.50	FFLC	0.045
Aminoimidazole ribonucleotide biosynthesis I	−0.50	FFLC	0.046
UDP *N*-acetylmuramoyl pentapeptide biosynthesis I meso diaminopimelate containing	−0.49	FFLC	0.049
Thiamin salvage II	−0.49	FFLC	0.049
Glycolysis II from fructose 6-phosphate	−0.45	FFLC	0.049
Purine nucleotides degradation II aerobic	0.54	FFLC	0.027
Guanosine nucleotides degradation III	0.54	FFLC	0.025
Adenosine nucleotides degradation II	0.56	FFLC	0.019
Purine nucleobases degradation I anaerobic	0.56	FFLC	0.018
Anhydromuropeptides recycling	−0.69	HF	0.001
TCA cycle VI obligate autotrophs	−0.67	HF	0.003
TCA cycle I prokaryotic	−0.66	HF	0.003
Reductive TCA cycle I	−0.64	HF	0.005
tRNA processing	−0.63	HF	0.005
Superpathway of fucose and rhamnose degradation	0.62	HF	0.007
Arginine ornithine and proline interconversion	0.59	HF	0.012
l-rhamnose degradation I	0.57	HF	0.017
TCA cycle V 2-oxoglutarate ferredoxin oxidoreductase	−0.54	HF	0.024
Superpathway of l-alanine biosynthesis	−0.54	HF	0.026
Fucose degradation	0.52	HF	0.035
l-lysine fermentation to acetate and butanoate	0.51	HF	0.040

ρ Spearman’s rho correlation.

**Table 3:** Significant correlations between bacterial metabolic pathway abundance and fasting cholesterol.

Predicted Metabolic Pathways	ρ	Diet	AdjP
Glycolysis II from fructose 6-phosphate	−0.44	FFLC	0.049
Superpathway of L-aspartate and L-asparagine biosynthesis	−0.47	FFLC	0.034
Teichoic acid poly glycerol biosynthesis	−0.49	FFLC	0.050

ρ Spearman’s rho correlation.

## Supplementary Materials

The following are available online at https://www.mdpi.com/2072-6643/12/11/3557/s1, Supplementary Figure S1. Significant differences in predicted microbial metabolic pathway abundances between dietary interventions with combined measurements taken at day 7 and day 14. Two-sided permutation t-test, with multiple comparison correction with FDR Value expressed as absolute relative change; colors denote significant increases for each dietary intervention study group. FFLC denotes a high-fat, high-fructose diet, HF a low-fat/high-fructose diet, and FDR multiple correction for false discovery rate. Supplementary Table S1. Dietary components of customized experimental diets: high-fat/high fructose (FFLC) and high-fructose/low fat (HF).

## References

[1] Turnbaugh P.J., Hamady M., Yatsunenko T., Cantarel B.L., Duncan A., Ley R.E., Sogin M.L., Jones W.J., Roe B.A., Affourtit J.P. (2009). A core gut microbiome in obese and lean twins. Nature.

[2] Hooper L.V., MacPherson A.J. (2010). Immune adaptations that maintain homeostasis with the intestinal microbiota. Nat. Rev. Immunol..

[3] Koppel N., Maini Rekdal V., Balskus E.P. (2017). Chemical transformation of xenobiotics by the human gut microbiota. Science.

[4] Durack J., Lynch S.V. (2019). The gut microbiome: Relationships with disease and opportunities for therapy. J. Exp. Med..

[5] McBurney M.I., Davis C., Fraser C.M., Schneeman B.O., Huttenhower C., Verbeke K., Walter J., Latulippe M.E. (2019). Establishing What Constitutes a Healthy Human Gut Microbiome: State of the Science, Regulatory Considerations, and Future Directions. J. Nutr..

[6] Qin J., Li Y., Cai Z., Li S., Zhu J., Zhang F., Liang S., Zhang W., Guan Y., Shen D. (2012). A metagenome-wide association study of gut microbiota in type 2 diabetes. Nature.

[7] Henao-Mejia J., Elinav E., Jin C., Hao L., Mehal W.Z., Strowig T., Thaiss C.A., Kau A.L., Eisenbarth S.C., Jurczak M.J. (2012). Inflammasome-mediated dysbiosis regulates progression of NAFLD and obesity. Nature.

[8] Ley R.E., Backhed F., Turnbaugh P., Lozupone C.A., Knight R.D., Gordon J.I. (2005). Obesity alters gut microbial ecology. Proc. Natl. Acad. Sci. USA.

[9] Ley R.E., Turnbaugh P.J., Klein S., Gordon J.I. (2006). Microbial ecology: Human gut microbes associated with obesity. Nature.

[10] Lee L., Sanders R.A. (2012). Metabolic syndrome. Pediatr. Rev..

[11] Kenny D.J., Plichta D.R., Shungin D., Koppel N., Hall A.B., Fu B., Vasan R.S., Shaw S.Y., Vlamakis H., Balskus E.P. (2020). Cholesterol Metabolism by Uncultured Human Gut Bacteria Influences Host Cholesterol Level. Cell Host Microbe.

[12] Cani P.D., Bibiloni R., Knauf C., Waget A., Neyrinck A.M., Delzenne N.M., Burcelin R. (2008). Changes in gut microbiota control metabolic endotoxemia-induced inflammation in high-fat diet-induced obesity and diabetes in mice. Diabetes.

[13] Ijaz M.U., Ahmed M.I., Zou X., Hussain M., Zhang M., Zhao F., Xu X., Zhou G., Li C. (2018). Beef, casein, and soy proteins differentially affect lipid metabolism, triglycerides accumulation and gut microbiota of high-fat diet-fed C57BL/6J mice. Front. Microbiol..

[14] Lozano I., Van der Werf R., Bietiger W., Seyfritz E., Peronet C., Pinget M., Jeandidier N., Maillard E., Marchioni E., Sigrist S. (2016). High-fructose and high-fat diet-induced disorders in rats: Impact on diabetes risk, hepatic and vascular complications. Nutr. Metab..

[15] Guerville M., Leroy A., Sinquin A., Laugerette F., Michalski M.C., Boudry G. (2017). Western-diet consumption induces alteration of barrier function mechanisms in the ileum that correlates with metabolic endotoxemia in rats. Am. J. Physiol. Endocrinol. Metab..

[16] Guevara-Cruz M., Flores-Lopez A.G., Aguilar-Lopez M., Sanchez-Tapia M., Medina-Vera I., Diaz D., Tovar A.R., Torres N. (2019). Improvement of Lipoprotein Profile and Metabolic Endotoxemia by a Lifestyle Intervention That Modifies the Gut Microbiota in Subjects With Metabolic Syndrome. J. Am. Heart Assoc..

[17] Roager H.M., Dragsted L.O. (2019). Diet-derived microbial metabolites in health and disease. Nutr. Bull..

[18] Jegatheesan P., De Bandt J.P. (2017). Fructose and NAFLD: The Multifaceted Aspects of Fructose Metabolism. Nutrients.

[19] Jegatheesan P., Beutheu S., Ventura G., Sarfati G., Nubret E., Kapel N., Waligora-Dupriet A.J., Bergheim I., Cynober L., De-Bandt J.P. (2016). Effect of specific amino acids on hepatic lipid metabolism in fructose-induced non-alcoholic fatty liver disease. Clin. Nutr..

[20] Oh J.H., Alexander L.M., Pan M., Schueler K.L., Keller M.P., Attie A.D., Walter J., van Pijkeren J.P. (2019). Dietary Fructose and Microbiota-Derived Short-Chain Fatty Acids Promote Bacteriophage Production in the Gut Symbiont Lactobacillus reuteri. Cell Host Microbe.

[21] Jakobsdottir G., Xu J., Molin G., Ahrne S., Nyman M. (2013). High-fat diet reduces the formation of butyrate, but increases succinate, inflammation, liver fat and cholesterol in rats, while dietary fibre counteracts these effects. PLoS ONE.

[22] Hoyles L., Fernandez-Real J.M., Federici M., Serino M., Abbott J., Charpentier J., Heymes C., Luque J.L., Anthony E., Barton R.H. (2018). Molecular phenomics and metagenomics of hepatic steatosis in non-diabetic obese women. Nat. Med..

[23] Ma N., Tian Y., Wu Y., Ma X. (2017). Contributions of the Interaction Between Dietary Protein and Gut Microbiota to Intestinal Health. Curr. Protein. Pept. Sci..

[24] Do T.T., Hindlet P., Waligora-Dupriet A.J., Kapel N., Neveux N., Mignon V., Delomenie C., Farinotti R., Feve B., Buyse M. (2014). Disturbed intestinal nitrogen homeostasis in a mouse model of high-fat diet-induced obesity and glucose intolerance. Am. J. Physiol. Endocrinol. Metab..

[25] Xiao F., Du Y., Lv Z., Chen S., Zhu J., Sheng H., Guo F. (2016). Effects of essential amino acids on lipid metabolism in mice and humans. J. Mol. Endocrinol..

[26] Hsieh J., Trajcevski K.E., Farr S.L., Baker C.L., Lake E.J., Taher J., Iqbal J., Hussain M.M., Adeli K. (2015). Glucagon-Like Peptide 2 (GLP-2) Stimulates Postprandial Chylomicron Production and Postabsorptive Release of Intestinal Triglyceride Storage Pools via Induction of Nitric Oxide Signaling in Male Hamsters and Mice. Endocrinology.

[27] Dalboge L.S., Pedersen P.J., Hansen G., Fabricius K., Hansen H.B., Jelsing J., Vrang N. (2015). A Hamster Model of Diet-Induced Obesity for Preclinical Evaluation of Anti-Obesity, Anti-Diabetic and Lipid Modulating Agents. PLoS ONE.

[28] Wang P.R., Guo Q., Ippolito M., Wu M., Milot D., Ventre J., Doebber T., Wright S.D., Chao Y.S. (2001). High fat fed hamster, a unique animal model for treatment of diabetic dyslipidemia with peroxisome proliferator activated receptor alpha selective agonists. Eur. J. Pharm..

[29] Bravo E., Cantafora A., Calcabrini A., Ortu G. (1994). Why prefer the golden Syrian hamster (Mesocricetus auratus) to the Wistar rat in experimental studies on plasma lipoprotein metabolism?. Comp. Biochem. Physiol. Part B Comp. Biochem..

[30] Gaynor B.J., Sand T., Clark R.W., Aiello R.J., Bamberger M.J., Moberly J.B. (1994). Inhibition of cholesteryl ester transfer protein activity in hamsters alters HDL lipid composition. Atherosclerosis.

[31] Nistor A., Bulla A., Filip D.A., Radu A. (1987). The hyperlipidemic hamster as a model of experimental atherosclerosis. Atherosclerosis.

[32] Shan K., Qu H., Zhou K., Wang L., Zhu C., Chen H., Gu Z., Cui J., Fu G., Li J. (2019). Distinct Gut Microbiota Induced by Different Fat-to-Sugar-Ratio High-Energy Diets Share Similar Pro-obesity Genetic and Metabolite Profiles in Prediabetic Mice. mSystems.

[33] Do M.H., Lee E., Oh M.J., Kim Y., Park H.Y. (2018). High-Glucose or -Fructose Diet Cause Changes of the Gut Microbiota and Metabolic Disorders in Mice without Body Weight Change. Nutrients.

[34] Whelan F.J., Verschoor C.P., Stearns J.C., Rossi L., Luinstra K., Loeb M., Smieja M., Johnstone J., Surette M.G., Bowdish D.M. (2014). The loss of topography in the microbial communities of the upper respiratory tract in the elderly. Ann. Am. Thorac. Soc..

[35] Horne R., St Pierre J., Odeh S., Surette M., Foster J.A. (2019). Microbe and host interaction in gastrointestinal homeostasis. Psychopharmacology.

[36] Martin M. (2011). Cutadapt removes adapter sequences from high-throughput sequencing reads. EMBnet J..

[37] Quast C., Pruesse E., Yilmaz P., Gerken J., Schweer T., Yarza P., Peplies J., Glockner F.O. (2013). The SILVA ribosomal RNA gene database project: Improved data processing and web-based tools. Nucleic Acids Res..

[38] Douglas G.M., Maffei V.J., Zaneveld J.R., Yurgel S.N., Brown J.R., Taylor C.M., Huttenhower C., Langille M.G.I. (2020). PICRUSt2 for prediction of metagenome functions. Nat. Biotechnol..

[39] Zhang X., Pei Y.F., Zhang L., Guo B., Pendegraft A.H., Zhuang W., Yi N. (2018). Negative Binomial Mixed Models for Analyzing Longitudinal Microbiome Data. Front. Microbiol..

[40] Fernandez-Mejia C., Lazo-de-la-Vega-Monroy M.-L. (2011). Biological Effects of Pharmacological Concentrations of Biotin. J. Evid. Based Integr. Med..

[41] Revilla-Monsalve C., Zendejas-Ruiz I., Islas-Andrade S., Baez-Saldana A., Palomino-Garibay M.A., Hernandez-Quiroz P.M., Fernandez-Mejia C. (2006). Biotin supplementation reduces plasma triacylglycerol and VLDL in type 2 diabetic patients and in nondiabetic subjects with hypertriglyceridemia. Biomed. Pharm..

[42] Higashimura Y., Naito Y., Takagi T., Uchiyama K., Mizushima K., Yoshikawa T. (2015). Propionate Promotes Fatty Acid Oxidation through the Up-Regulation of Peroxisome Proliferator-Activated Receptor alpha in Intestinal Epithelial Cells. J. Nutr. Sci. Vitam..

[43] Carr T.P., Weller C.L., Schlegel V.L., Cuppett S.L., Guderian D.M., Johnson K.R. (2005). Grain sorghum lipid extract reduces cholesterol absorption and plasma non-HDL cholesterol concentration in hamsters. J. Nutr..

[44] Martinez I., Wallace G., Zhang C., Legge R., Benson A.K., Carr T.P., Moriyama E.N., Walter J. (2009). Diet-induced metabolic improvements in a hamster model of hypercholesterolemia are strongly linked to alterations of the gut microbiota. Appl. Env. Microbiol..

[45] Kim H., Kim D.H., Seo K.H., Chon J.W., Nah S.Y., Bartley G.E., Arvik T., Lipson R., Yokoyama W. (2015). Modulation of the intestinal microbiota is associated with lower plasma cholesterol and weight gain in hamsters fed chardonnay grape seed flour. J. Agric. Food Chem..

[46] Pongking T., Haonon O., Dangtakot R., Onsurathum S., Jusakul A., Intuyod K., Sangka A., Anutrakulchai S., Cha’on U., Pinlaor S. (2020). A combination of monosodium glutamate and high-fat and high-fructose diets increases the risk of kidney injury, gut dysbiosis and host-microbial co-metabolism. PLoS ONE.

[47] Wang Y., Tong Q., Shou J.W., Zhao Z.X., Li X.Y., Zhang X.F., Ma S.R., He C.Y., Lin Y., Wen B.Y. (2017). Gut Microbiota-Mediated Personalized Treatment of Hyperlipidemia Using Berberine. Theranostics.

[48] Zinocker M.K., Lindseth I.A. (2018). The Western Diet-Microbiome-Host Interaction and Its Role in Metabolic Disease. Nutrients.

[49] Le K.A., Ith M., Kreis R., Faeh D., Bortolotti M., Tran C., Boesch C., Tappy L. (2009). Fructose overconsumption causes dyslipidemia and ectopic lipid deposition in healthy subjects with and without a family history of type 2 diabetes. Am. J. Clin. Nutr..

[50] Stanhope K.L., Schwarz J.M., Keim N.L., Griffen S.C., Bremer A.A., Graham J.L., Hatcher B., Cox C.L., Dyachenko A., Zhang W. (2009). Consuming fructose-sweetened, not glucose-sweetened, beverages increases visceral adiposity and lipids and decreases insulin sensitivity in overweight/obese humans. J. Clin. Investig..

[51] Softic S., Gupta M.K., Wang G.X., Fujisaka S., O’Neill B.T., Rao T.N., Willoughby J., Harbison C., Fitzgerald K., Ilkayeva O. (2018). Divergent effects of glucose and fructose on hepatic lipogenesis and insulin signaling. J. Clin. Investig..

[52] Tillman E.J., Morgan D.A., Rahmouni K., Swoap S.J. (2014). Three months of high-fructose feeding fails to induce excessive weight gain or leptin resistance in mice. PLoS ONE.

[53] Messier C., Whately K., Liang J., Du L., Puissant D. (2007). The effects of a high-fat, high-fructose, and combination diet on learning, weight, and glucose regulation in C57BL/6 mice. Behav. Brain Res..

[54] Simonson D.C., Tappy L., Jequier E., Felber J.P., DeFronzo R.A. (1988). Normalization of carbohydrate-induced thermogenesis by fructose in insulin-resistant states. Am. J. Physiol..

[55] Mizobe T., Nakajima Y., Ueno H., Sessler D.I. (2006). Fructose administration increases intraoperative core temperature by augmenting both metabolic rate and the vasoconstriction threshold. Anesthesiology.

[56] DeBosch B.J., Chen Z., Finck B.N., Chi M., Moley K.H. (2013). Glucose transporter-8 (GLUT8) mediates glucose intolerance and dyslipidemia in high-fructose diet-fed male mice. Mol. Endocrinol..

[57] Taghibiglou C., Carpentier A., Van Iderstine S.C., Chen B., Rudy D., Aiton A., Lewis G.F., Adeli K. (2000). Mechanisms of hepatic very low density lipoprotein overproduction in insulin resistance. Evidence for enhanced lipoprotein assembly, reduced intracellular ApoB degradation, and increased microsomal triglyceride transfer protein in a fructose-fed hamster model. J. Biol Chem..

[58] Leeming E.R., Johnson A.J., Spector T.D., Le Roy C.I. (2019). Effect of Diet on the Gut Microbiota: Rethinking Intervention Duration. Nutrients.

[59] Voreades N., Kozil A., Weir T.L. (2014). Diet and the development of the human intestinal microbiome. Front. Microbiol..

[60] Wei S., Mortensen M.S., Stokholm J., Brejnrod A.D., Thorsen J., Rasmussen M.A., Trivedi U., Bisgaard H., Sorensen S.J. (2018). Short- and long-term impacts of azithromycin treatment on the gut microbiota in children: A double-blind, randomized, placebo-controlled trial. EBioMedicine.

[61] Magne F., Gotteland M., Gauthier L., Zazueta A., Pesoa S., Navarrete P., Balamurugan R. (2020). The Firmicutes/Bacteroidetes Ratio: A Relevant Marker of Gut Dysbiosis in Obese Patients?. Nutrients.

[62] Murugesan S., Ulloa-Martinez M., Martinez-Rojano H., Galvan-Rodriguez F.M., Miranda-Brito C., Romano M.C., Pina-Escobedo A., Pizano-Zarate M.L., Hoyo-Vadillo C., Garcia-Mena J. (2015). Study of the diversity and short-chain fatty acids production by the bacterial community in overweight and obese Mexican children. Eur. J. Clin. Microbiol. Infect. Dis..

[63] Lin H., An Y., Tang H., Wang Y. (2019). Alterations of Bile Acids and Gut Microbiota in Obesity Induced by High Fat Diet in Rat Model. J. Agric. Food Chem..

[64] Zhao L., Chen Y., Xia F., Abudukerimu B., Zhang W., Guo Y., Wang N., Lu Y. (2018). A Glucagon-Like Peptide-1 Receptor Agonist Lowers Weight by Modulating the Structure of Gut Microbiota. Front. Endocrinol..

[65] Hiippala K., Kainulainen V., Suutarinen M., Heini T., Bowers J.R., Jasso-Selles D., Lemmer D., Valentine M., Barnes R., Engelthaler D.M. (2020). Isolation of Anti-Inflammatory and Epithelium Reinforcing Bacteroides and Parabacteroides Spp. from A Healthy Fecal Donor. Nutrients.

[66] Wang K., Liao M., Zhou N., Bao L., Ma K., Zheng Z., Wang Y., Liu C., Wang W., Wang J. (2019). Parabacteroides distasonis Alleviates Obesity and Metabolic Dysfunctions via Production of Succinate and Secondary Bile Acids. Cell Rep..

[67] Ridaura V.K., Faith J.J., Rey F.E., Cheng J., Duncan A.E., Kau A.L., Griffin N.W., Lombard V., Henrissat B., Bain J.R. (2013). Gut microbiota from twins discordant for obesity modulate metabolism in mice. Science.

[68] Alang N., Kelly C.R. (2015). Weight gain after fecal microbiota transplantation. Open Forum Infect. Dis.

[69] Kelly T.N., Bazzano L.A., Ajami N.J., He H., Zhao J., Petrosino J.F., Correa A., He J. (2016). Gut Microbiome Associates with Lifetime Cardiovascular Disease Risk Profile among Bogalusa Heart Study Participants. Circ. Res..

[70] Kuang Y.S., Lu J.H., Li S.H., Li J.H., Yuan M.Y., He J.R., Chen N.N., Xiao W.Q., Shen S.Y., Qiu L. (2017). Connections between the human gut microbiome and gestational diabetes mellitus. Gigascience.

[71] Rossell J., Brindefalk B., Baena-Fustegueras J.A., Peinado-Onsurbe J., Udekwu K.I. (2020). Diet change affects intestinal microbiota restoration and improves vertical sleeve gastrectomy outcome in diet-induced obese rats. Eur. J. Nutr..

[72] Aguilera-Mendez A., Hernandez-Equihua M.G., Rueda-Rocha A.C., Guajardo-Lopez C., Nieto-Aguilar R., Serrato-Ochoa D., Ruiz Herrera L.F., Guzman-Nateras J.A. (2018). Protective effect of supplementation with biotin against high-fructose-induced metabolic syndrome in rats. Nutr. Res..

[73] Nakagawa T., Hu H., Zharikov S., Tuttle K.R., Short R.A., Glushakova O., Ouyang X., Feig D.I., Block E.R., Herrera-Acosta J. (2006). A causal role for uric acid in fructose-induced metabolic syndrome. Am. J. Physiol. Ren. Physiol..

[74] Lanaspa M.A., Sanchez-Lozada L.G., Choi Y.J., Cicerchi C., Kanbay M., Roncal-Jimenez C.A., Ishimoto T., Li N., Marek G., Duranay M. (2012). Uric acid induces hepatic steatosis by generation of mitochondrial oxidative stress: Potential role in fructose-dependent and -independent fatty liver. J. Biol. Chem..

[75] Cherbuy C., Bellet D., Robert V., Mayeur C., Schwiertz A., Langella P. (2019). Modulation of the Caecal Gut Microbiota of Mice by Dietary Supplement Containing Resistant Starch: Impact Is Donor-Dependent. Front. Microbiol..

[76] Pelgrim C.E., Franx B.A.A., Snabel J., Kleemann R., Arnoldussen I.A.C., Kiliaan A.J. (2017). Butyrate Reduces HFD-Induced Adipocyte Hypertrophy and Metabolic Risk Factors in Obese LDLr-/-.Leiden Mice. Nutrients.

[77] Yan H., Ajuwon K.M. (2015). Mechanism of Butyrate Stimulation of Triglyceride Storage and Adipokine Expression during Adipogenic Differentiation of Porcine Stromovascular Cells. PLoS ONE.

[78] Yu C., Liu S., Chen L., Shen J., Niu Y., Wang T., Zhang W., Fu L. (2019). Effect of exercise and butyrate supplementation on microbiota composition and lipid metabolism. J. Endocrinol..

[79] Chambers E.S., Viardot A., Psichas A., Morrison D.J., Murphy K.G., Zac-Varghese S.E., MacDougall K., Preston T., Tedford C., Finlayson G.S. (2015). Effects of targeted delivery of propionate to the human colon on appetite regulation, body weight maintenance and adiposity in overweight adults. Gut.

[80] Preiss B. (1985). Regulation of HMG-CoA Reductase in Extrahepatic Tissues. Regul. Hmg-Coa Reductase.

[81] Greenspan M.D., Yudkovitz J.B., Lo C.Y., Chen J.S., Alberts A.W., Hunt V.M., Chang M.N., Yang S.S., Thompson K.L., Chiang Y.C. (1987). Inhibition of hydroxymethylglutaryl-coenzyme A synthase by L-659,699. Proc. Natl. Acad. Sci. USA.

[82] Hegardt F.G. (1999). Mitochondrial 3-hydroxy-3-methylglutaryl-CoA synthase: A control enzyme in ketogenesis. Biochem. J..

[83] Alex S., Lange K., Amolo T., Grinstead J.S., Haakonsson A.K., Szalowska E., Koppen A., Mudde K., Haenen D., Al-Lahham S. (2013). Short-chain fatty acids stimulate angiopoietin-like 4 synthesis in human colon adenocarcinoma cells by activating peroxisome proliferator-activated receptor gamma. Mol. Cell Biol..

[84] Byndloss M.X. (2020). Microbial management. Science.

[85] Ottosson F., Smith E., Melander O., Fernandez C. (2018). Altered Asparagine and Glutamate Homeostasis Precede Coronary Artery Disease and Type 2 Diabetes. J. Clin. Endocrinol. Metab..

[86] Yanni A.E., Agrogiannis G., Nomikos T., Fragopoulou E., Pantopoulou A., Antonopoulou S., Perrea D. (2010). Oral supplementation with L-aspartate and L-glutamate inhibits atherogenesis and fatty liver disease in cholesterol-fed rabbit. Amino Acids.

[87] Kujala U.M., Makinen V.P., Heinonen I., Soininen P., Kangas A.J., Leskinen T.H., Rahkila P., Wurtz P., Kovanen V., Cheng S. (2013). Long-term leisure-time physical activity and serum metabolome. Circulation.

[88] Wang T.J., Larson M.G., Vasan R.S., Cheng S., Rhee E.P., McCabe E., Lewis G.D., Fox C.S., Jacques P.F., Fernandez C. (2011). Metabolite profiles and the risk of developing diabetes. Nat. Med..

[89] Kujala U.M., Peltonen M., Laine M.K., Kaprio J., Heinonen O.J., Sundvall J., Eriksson J.G., Jula A., Sarna S., Kainulainen H. (2016). Branched-Chain Amino Acid Levels Are Related with Surrogates of Disturbed Lipid Metabolism among Older Men. Front. Med..

