# Supplementary material for: High Fat-High Fructose Diet-Induced Changes in the Gut Microbiota Associated with Dyslipidemia in Syrian Hamsters
Source: Nutrients. 2020 Nov 20;12(11):3557. doi: 10.3390/nu12113557 (PMC7699731; doi:10.3390/nu12113557)
Supplement: Supplementary file 1 [file nutrients-12-03557-s001.pdf]

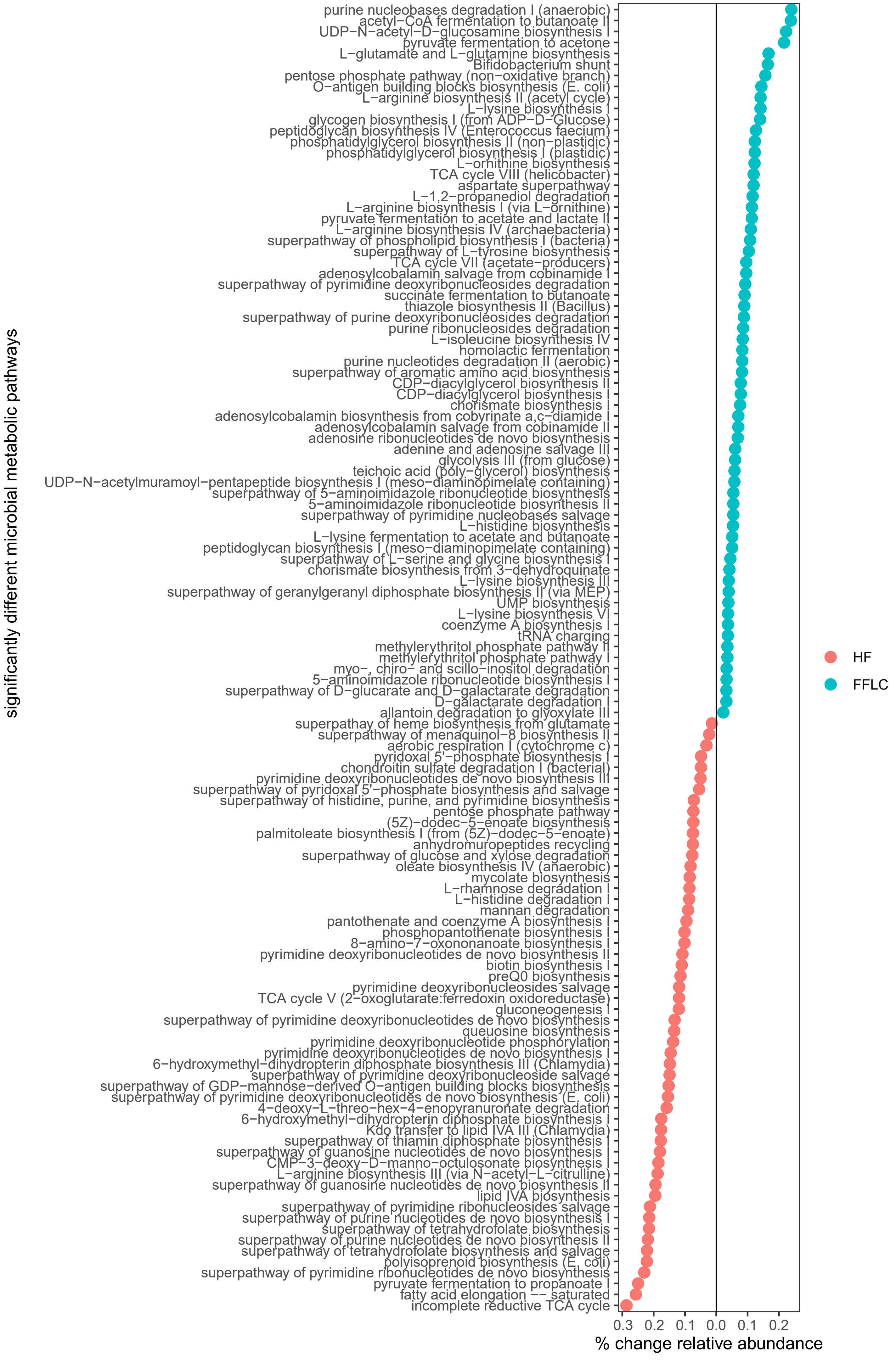

purine nucleobases degradation I (anaerobic)
acetyl−CoA fermentation to butanoate II
UDP−N−acetyl−D−glucosamine biosynthesis I
pyruvate fermentation to acetone
L−glutamate and L−glutamine biosynthesis
Bifidobacterium shunt
pentose phosphate pathway (non−oxidative branch)
O−antigen building blocks biosynthesis (E. coli)
L−arginine biosynthesis II (acetyl cycle)
L−lysine biosynthesis I
glycogen biosynthesis I (from ADP−D−Glucose)
peptidoglycan biosynthesis IV (Enterococcus faecium)
phosphatidylglycerol biosynthesis II (non−plastidic)
phosphatidylglycerol biosynthesis I (plastidic)
L−ornithine biosynthesis
TCA cycle VIII (helicobacter)
aspartate superpathway
L−1,2−propanediol degradation
L−arginine biosynthesis I (via L−ornithine)
pyruvate fermentation to acetate and lactate II
L−arginine biosynthesis IV (archaebacteria)
superpathway of phospholipid biosynthesis I (bacteria)
superpathway of L−tyrosine biosynthesis
TCA cycle VII (acetate−producers)
adenosylcobalamin salvage from cobinamide I
superpathway of pyrimidine deoxyribonucleosides degradation
succinate fermentation to butanoate
thiazole biosynthesis II (Bacillus)
superpathway of purine deoxyribonucleosides degradation
purine ribonucleosides degradation
L−isoleucine biosynthesis IV
homolactic fermentation
purine nucleotides degradation II (aerobic)
superpathway of aromatic amino acid biosynthesis
CDP−diacylglycerol biosynthesis II
CDP−diacylglycerol biosynthesis I
chorismate biosynthesis I
adenosylcobalamin biosynthesis from cobyrinate a,c−diamide I
adenosylcobalamin salvage from cobinamide II
adenosine ribonucleotides de novo biosynthesis
adenine and adenosine salvage III
glycolysis III (from glucose)
teichoic acid (poly−glycerol) biosynthesis
UDP−N−acetylmuramoyl−pentapeptide biosynthesis I (meso−diaminopimelate containing)
superpathway of 5−aminoimidazole ribonucleotide biosynthesis
5−aminoimidazole ribonucleotide biosynthesis II
superpathway of pyrimidine nucleobases salvage
L−histidine biosynthesis
L−lysine fermentation to acetate and butanoate
peptidoglycan biosynthesis I (meso−diaminopimelate containing)
superpathway of L−serine and glycine biosynthesis I
chorismate biosynthesis from 3−dehydroquinate
L−lysine biosynthesis III
superpathway of geranylgeranyl diphosphate biosynthesis II (via MEP)
UMP biosynthesis
L−lysine biosynthesis VI
coenzyme A biosynthesis I
tRNA charging
methylerythritol phosphate pathway II
methylerythritol phosphate pathway I
myo−, chiro− and scillo−inositol degradation
5−aminoimidazole ribonucleotide biosynthesis I
superpathway of D−glucarate and D−galactarate degradation
D−galactarate degradation I
allantoin degradation to glyoxylate III
superpathay of heme biosynthesis from glutamate
superpathway of menaquinol−8 biosynthesis II
aerobic respiration I (cytochrome c)
pyridoxal 5'−phosphate biosynthesis I
chondroitin sulfate degradation I (bacterial)
pyrimidine deoxyribonucleotides de novo biosynthesis III
superpathway of pyridoxal 5'−phosphate biosynthesis and salvage
superpathway of histidine, purine, and pyrimidine biosynthesis
pentose phosphate pathway
(5Z)−dodec−5−enoate biosynthesis
palmitoleate biosynthesis I (from (5Z)−dodec−5−enoate)
anhydromuropeptides recycling
superpathway of glucose and xylose degradation
oleate biosynthesis IV (anaerobic)
mycolate biosynthesis
L−rhamnose degradation I
L−histidine degradation I
mannan degradation
pantothenate and coenzyme A biosynthesis I
phosphopantothenate biosynthesis I
8−amino−7−oxononanoate biosynthesis I
pyrimidine deoxyribonucleotides de novo biosynthesis II
biotin biosynthesis I
preQ0 biosynthesis
pyrimidine deoxyribonucleosides salvage
TCA cycle V (2−oxoglutarate:ferredoxin oxidoreductase)
gluconeogenesis I
superpathway of pyrimidine deoxyribonucleotides de novo biosynthesis
queuosine biosynthesis
pyrimidine deoxyribonucleotide phosphorylation
pyrimidine deoxyribonucleotides de novo biosynthesis I
6−hydroxymethyl−dihydropterin diphosphate biosynthesis III (Chlamydia)
superpathway of pyrimidine deoxyribonucleoside salvage
superpathway of GDP−mannose−derived O−antigen building blocks biosynthesis
superpathway of pyrimidine deoxyribonucleotides de novo biosynthesis (E. coli)
4−deoxy−L−threo−hex−4−enopyranuronate degradation
6−hydroxymethyl−dihydropterin diphosphate biosynthesis I
Kdo transfer to lipid IVA III (Chlamydia)
superpathway of thiamin diphosphate biosynthesis I
superpathway of guanosine nucleotides de novo biosynthesis I
CMP−3−deoxy−D−manno−octulosonate biosynthesis I
L−arginine biosynthesis III (via N−acetyl−L−citrulline)
superpathway of guanosine nucleotides de novo biosynthesis II
lipid IVA biosynthesis
superpathway of pyrimidine ribonucleosides salvage
superpathway of purine nucleotides de novo biosynthesis I
superpathway of tetrahydrofolate biosynthesis
superpathway of purine nucleotides de novo biosynthesis II
superpathway of tetrahydrofolate biosynthesis and salvage
polyisoprenoid biosynthesis (E. coli)
superpathway of pyrimidine ribonucleotides de novo biosynthesis
pyruvate fermentation to propanoate I
fatty acid elongation −− saturated
incomplete reductive TCA cycle
significantly different microbial metabolic pathways
0.3
0.2
0.1
0.0
0.1
0.2
% change relative abundance
HF
FFLC

Supplemental Table 1. Dietary components of customized experimental diets: high-fat/high fructose (FFLC) and high-fructose/low fat (HF).

Dietary Component	kcal/kg		g/kg	
	FFLC	HF	FFLC	HF
**Casein**	602.87	787.6	168.4	220
**L-Arginine**	4.88	4	1.22	1
**L-Tryptophan**	5.368	4.4	1.34	1.1
**Fructose**	1572.42	2280	413.79	600
**Cellulose**	0	0	58.5	70.9
**Corn oil**	472.86	540	52.54	60
**Beef Tallow**	2227.14	0	247.46	0
**Mineral mix #260001**	37.57	0	42.7	35
**Vitamin mix #360001**	47.58	39.4	12.2	10
**Choline Bitartrate**	0	0	1.342	2
**Cholesterol**	0	0	0.5	0
**Total**	4970.7	3655.4	1000	1000

Control diet (Chow) represents the PicoLab Mouse diet 20 5058* which is a proprietary diet. The product datasheet can be found at:

https://www.labdiet.com/cs/groups/lolweb/@labdiet/documents/web_content/mdrf/mdi4/~edisp/ducm04_028435.pdf
